# Investigating the clinical predictive utility of inflammatory markers and nomogram development in colorectal cancer patients with malnutrition

**DOI:** 10.3389/fnut.2024.1442094

**Published:** 2024-11-26

**Authors:** Xuexing Wang, Xingxing Tang, Jinsong Xu, Rong Zhang, Jie Chu, Chunmei Chen, Chunmei Wei

**Affiliations:** ^1^Department of Oncology, Anning First People's Hospital Affiliated to Kunming University of Science and Technology, Kunming, China; ^2^Department of Thoracic Surgery, The Third People's Hospital of Honghe Hani and Yi Autonomous Prefecture, Honghe, China; ^3^Department of Geriatric Oncology, The Third Affiliated Hospital of Kunming Medical University, Yunnan Cancer Hospital, Kunming, China; ^4^Department of Oncology, Ziyang Central Hospital, Ziyang, China; ^5^Department of Pharmacy, Anning First People’s Hospital Affiliated to Kunming University of Science and Technology, Kunming, China

**Keywords:** systemic immune-inflammatory, malnutrition, colorectal neoplasms, nutrition assessment, nomograms

## Abstract

**Objective:**

The aim of this study is to investigate the relationship and prognostic significance of serum neutrophil-lymphocyte ratio (NLR), systemic immune-inflammatory index (SII), platelet-lymphocyte ratio (PLR), and prognostic nutritional index (PNI) in colorectal cancer (CRC) patients with malnutrition, as well as to construct a nomogram for predicting the onset of malnutrition.

**Methods:**

The clinical data of 391 inpatients who were hospitalized from December 1, 2021 to January 31, 2023 the diagnosis of CRC were selected and divided into a malnutrition group (121 cases) and a well-nourished group (270 cases) according to whether they were malnourished or not. Focusing on comparing the differences in serum NLR, PLR, SII index, PNI index and general information between the two groups, the Binary logistics regression analysis was used to analyze the factors affecting malnutrition, and receiver operating characteristic (ROC) curves were established to assess the predictive value of serum NLR, PLR, SII index, and PNI index individually and jointly for malnutrition, and to calculate the optimal predictive thresholds. Finally a highly accurate clinical predictive nomogram was constructed.

**Results:**

Compared with the well-nourished group, the malnourished group had higher serum NLR, SII index, PLR and lower PNI index levels, with statistically significant differences (*p* < 0.001). The area under the curve of NLR, SII index, PLR, and PNI index alone and in combination predicted a poor prognosis of 0.705, 0.665, 0.636, 0.773, and 0.784, respectively. After conducting Logistic regression analysis, the nomogram, which included BMI, NRS-2002, long-term bed rest, and PNI, demonstrated strong predictive capabilities. Decision curves highlighted the clinical utility of the predictive nomograms. The receiver operating characteristic curve revealed strong discrimination (area under the curve [AUC] = 0.958, 95% CI: 0.937–0.979). Additionally, the ROC analysis indicated a sensitivity of 0.843 and specificity of 0.937. Calibration curves exhibited excellent concordance between nomogram predictions and observed outcomes. Decision curves highlighted the clinical utility of the predictive nomograms.

**Conclusion:**

Serum NLR, SII index, PLR, and PNI are significant predictive factors for the development of malnutrition in patients with CRC. These indices, whether considered individually or collectively, possess clinical relevance in forecasting malnutrition. Furthermore, the creation of an innovative nomogram prediction model offers considerable clinical utility.

## 1 Background

Colorectal cancer is a prevalent malignant neoplasm in China, with increasing incidence and mortality rates. It currently ranks second in incidence and fourth in mortality among all malignant neoplasms in the country, presenting a significant public health concern that jeopardizes human life and well-being (1). Malnutrition prevalence is higher among colorectal cancer patients compared to those with non-gastrointestinal cancers, attributed to increased consumption resulting from oncological factors and reduced intestinal absorption, with malnutrition rates ranging from 20 to 37% (2). Malnutrition has been shown to have detrimental effects on various aspects of patient outcomes in colorectal cancer, including delayed surgical wound healing, heightened treatment-related immunosuppression, increased susceptibility to infectious complications, diminished quality of life, prolonged hospital stays, reduced treatment tolerance, and heightened treatment-related toxicity (3–5). Therefore, accurate evaluation of nutritional status in colorectal cancer patients is crucial for predicting unfavorable prognostic outcomes. The relationship between inflammation and malnutrition is intricate and increasingly recognized in academic literature (6). Numerous studies have demonstrated that inflammation plays a significant role in the development of malnutrition across various diseases. In tumor-related diseases, chronic low-grade inflammation is believed to be a primary contributor, impacting nociceptors and appetite through mechanisms such as inducing muscle breakdown due to rhabdomyolysis, impeding gastric emptying, and modulating hunger-related hormones. These processes can ultimately contribute to the onset of malnutrition (7, 8). Alternatively, certain academics posit that in the presence of inflammatory stimuli, immune and tumor cells secrete various inflammatory mediators such as interleukin-1β, interleukin-6, and tumor necrosis factor (TNF), which have the potential to impede albumin production in hepatocytes, consequently contributing to the onset of malnutrition (9–11).

Inflammation can be assessed through the enumeration of inflammatory cells, such as neutrophils, lymphocytes, and monocytes, in hematology. The neutrophil-to-lymphocyte ratio (NLR) is a metric used to quantify inflammation, and previous research has shown its utility in predicting malnutrition in elderly individuals (5, 12). The Systemic Immune Inflammatory Index (SII) is a composite index derived from the Neutrophil-to-Lymphocyte Ratio (NLR) and platelet count, commonly utilized in research as a significant prognostic indicator for cancer (13–15). The Platelet-to-Lymphocyte Ratio (PLR) serves as a marker reflecting platelet aggregation and systemic inflammation, facilitating the evaluation of inflammation-induced platelet activation, coagulation response, severe coagulation disorders, and systemic inflammatory response. Prior investigations have suggested that PLR could potentially serve as an independent risk factor for Stroke-Associated Pneumonia (SAP) in stroke patients (16). Prognostic Nutritional Index (PNI) has been considered in many studies as an independent predictor of poor outcome in patients with cancer, cardiovascular disease, and others (17, 18).

Currently, there is a scarcity of data regarding the correlation between NLR, SII index, PLR, and PNI with nutritional status in individuals diagnosed with colorectal cancer. Thus, the aim of this research was to investigate the association and predictive value of these parameters individually or in conjunction with nutritional status in individuals diagnosed with colorectal cancer, as well as to develop a nomogram for forecasting the likelihood of malnutrition.

## 2 Materials and methods

### 2.1 Participants and study design

This retrospective cohort study was conducted at the Anning First People’s Hospital Affiliated to Kunming University of Science and Technology (Kunming No. 4 People’s Hospital) in Kunming, Yunnan Province, China. The study included consecutive colorectal cancer patients admitted from December 1, 2021 to January 31, 2023, who met the specified inclusion criteria. Approval for the study protocol was obtained from the Ethics Committee of the Anning First People’s Hospital Affiliated to Kunming University of Science and Technology (Approval No. Lun Audit 2024-020). Furthermore, the research adhered to the guidelines outlined in the Declaration of Helsinki and was approved by the Ethics Committee. Informed consent was waived for this retrospective clinical research analysis as all sensitive data was anonymized, thus negating the need for patient consent.

**Figure 1:**
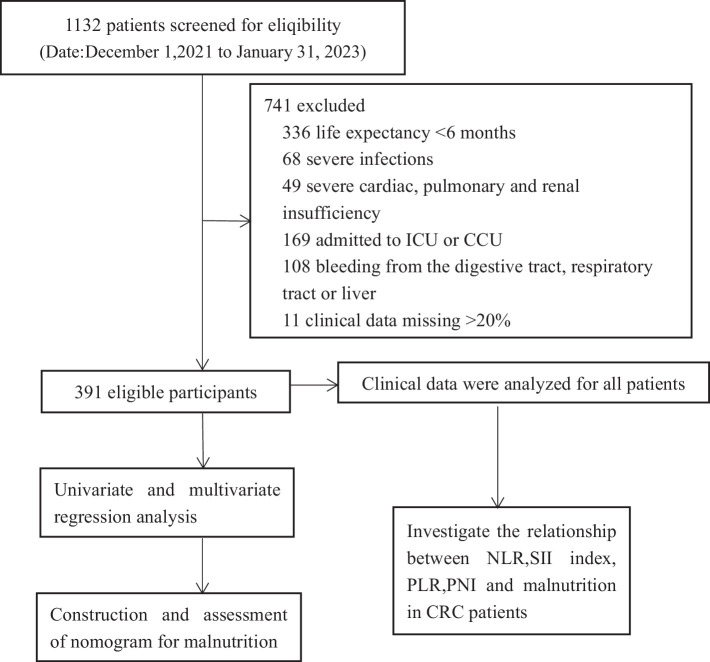
Experimental roadmap for study. This flow diagram indicates the workflow of the method present in this study.

Patients meeting the specified criteria were included in the study: (1) Hospitalized individuals diagnosed with stage I–IV colorectal cancer through pathological examination and imaging studies. (2) Patients not initially diagnosed with malnutrition upon admission, but who subsequently developed malnutrition within 3 months. (3) Enrolled patients possessed a Karnofsky Performance Status (KPS) score of 60 or higher. Patients meeting the specified criteria were excluded from the study, including those with an expected survival time of less than 6 months, individuals with severe infections or severe cardiac, pulmonary, and renal insufficiency, patients referred to the intensive care unit or cardiovascular care unit due to their condition, individuals experiencing combined bleeding from vital organs such as the digestive tract, respiratory tract, or liver, and patients with more than 20% missing data in the required case data. The study flow chart is shown in Figure 1.

### 2.2 Data collection

Data entry is done using a two-person validation system, a one-person entry and one-person validation system to ensure that the data is correct. Furthermore, given that the diagnostic criteria for malnutrition encompass alterations in pertinent indicators, a retrospective review of patients’ medical records from prior hospitalizations was undertaken by one of the investigators. This review primarily focused on nursing records to assess changes in weight, muscle measurements, and other relevant data. The use of the hospital information system (HIS) mainly captured general clinical information from patient cases mainly including the patient’s gender, household status, marital status, whether they were bedridden for a long period of time, age, KPS score, tumor stage, Body Mass Index (BMI), history of alcohol consumption, history of gastrointestinal surgeries (within the last 2 years), hypertension, diabetes mellitus, and coronary artery disease, nutritional risk screening 2002 (NRS-2002); laboratory tests: blood cell analysis: red blood cells (RBC), white blood cells (WBC), hemoglobin (HGB), platelets (PLT), neutrophils (NEUT), lymphocytes (LYM), albumin (ALB), pre-albumin (PAB), alanine aminotransferase (ALT), glutamate aminotransferase (AST), urea and serum creatinine values (Cr). The calculation of BMI, NLR, PLR, SII index, and PNI is performed using the following formulas: BMI is determined by dividing body weight in kilograms by height in meters squared; NLR is calculated as the ratio of neutrophil count to lymphocyte count; SII index is obtained by multiplying the neutrophil count by the platelet count and then dividing by the lymphocyte count; PLR is determined by dividing the platelet count by the lymphocyte count; and PNI is calculated by adding the serum albumin level to five times the lymphocyte count.

### 2.3 Determination and grouping of malnutrition

In this research, the diagnostic criteria for malnutrition utilized were the global leadership initiative on malnutrition criteria (GLIM) (19). The primary approach involved defining malnutrition risk as a score of ≥3 on the Nutritional Risk Screening 2002 (NRS-2002) tool, which was initially assessed by reviewing medical records. Subsequently, a second step was undertaken, which involved a detailed analysis of patient case data to assess malnutrition. Malnutrition was evaluated through three manifestations: involuntary weight loss (>5% within past 6 months or >10% beyond 6 months), low BMI [Asian standard: BMI <18.5 kg/m^2^ (<70 years), <20 kg/m^2^ (≥70 years)], decreased muscle mass (Decreased muscle mass is defined as a clinical condition characterized by a reduction in muscle mass and functional decline, typically diagnosed through by validated body composition techniques), and two etiologic indicators: decreased food intake or absorption (<50% intake for more than 1 week, or reduced intake for >2 weeks, or chronic gastrointestinal dysfunction, disease burden/inflammation). Malnutrition was evaluated based on the presence of at least one diagnostic criterion for manifestation and one diagnostic criterion for etiology. Patients were then classified into either malnourished or well-nourished group according to the final assessment.

### 2.4 Statistical analysis

Data entry was conducted using Excel 2019, while statistical analysis was performed using IBM SPSS Statistics for Windows version 26.0 (IBM Corp, Armonk, NY, USA). The normality of data distribution was evaluated using the Shapiro–Wilk test. Measurement data that followed a normal distribution were presented as mean ± standard deviation (*x* ± *s*) and compared using a t-test for two-group comparisons. Non-normally distributed data were presented as median (25th–75th percentile) and compared using the rank sum test. Count data were presented as [*n* (%)] and compared using the chi-square test for two-group comparisons.

To evaluate the effect of risk factors on outcomes, we first conducted correlation analysis followed by univariate regression analysis. Statistically significant variables from the univariate analysis (*p* < 0.05) were then included in the forward stepwise logistic regression analysis to identify independent predictors. The area under the curve (AUC) was calculated from the receiver operating characteristic (ROC) curve to assess predictive capability, with a higher AUC indicating better discriminatory capacity of the model. This analysis provides insight into the model’s ability to differentiate between malnourished and well-nourished patients. Additionally, the Youden Index was utilized to optimize sensitivity and specificity, thereby determining cut-off values for serum NLR, SII index, PLR, and PNI. Furthermore, the positive predictive value (PPV) and negative predictive value (NPV) were calculated based on the predictions of the logistic regression model, enabling us to assess the accuracy of diagnosing malnutrition among CRC patients. The performance of the nomogram was evaluated through the use of a concordance index (C-index) to measure the concordance between predicted probabilities and observed outcomes. One thousand bootstrap replications were conducted to assess the reliability of the results, with a higher C-index indicating more precise prognostic discrimination. Calibration curves were assessed visually, with excellent agreement defined as the predicted probabilities closely aligning with the observed outcomes, the calibration curve falling within the 95% confidence interval of the observed data, and a slope near 1. The total score for each patient in the validation cohort was computed based on the developed nomograms, followed by Cox regression analysis. Nomogram, C-index values, calibration curves, and decision curve analysis (DCA) were generated using the R software package (version 4.2.0).

## 3 Results

Our study ultimately included a total of 391 subjects, of whom 241 (61.6%) were male and 150 (38.4%) were female, with a mean age of 64.70 ± 11.72 years. Among them, Stage IV (57.3%), Urban households (62.7%), KPS scores ≥90 (54.2%) and History of surgery within 12 months (57.3%) were the most common. A total of 130 (33.2%) participants had NRS-2002 scores ≥3 and were at high nutritional risk. In the total sample, 121 (30.95%) patients with CRC were observed to be malnourished. The baseline characteristics of CRC patients are shown in Table 1. Table 1 illustrates statistically significant variations in Age, Prolonged bed rest, stage, KPS scores, Pain medication use, NRS-2002 scores, BMI, RBC, WBC, HGB, ALB, PAB, ALT, Urea, NLR, SII index, PLR, PNI between patients with malnutrition and well-nourished patients (*p* < 0.05).

**Table 1:** Demographic and clinical characteristics of the patients according to the nutritional status.

Characteristic	Total patients (*n* = 391)	Malnourished group (*n* = 121)	Well-nourished group (*n* = 270)	*p* value
Demographics				
Age (years), median [IQR]	66.00 [57.00,73.00]	69.00 [61.00, 76.00]	64.00 [54.00, 71.00]	<0.001
Sex, male, *n* (%)	241 (61.6)	75 (31.1)	166 (61.5)	0.509
Medical history				
Drinking, *n* (%)	46 (11.8)	18 (14.9)	28 (10.4)	0.134
Hypertension, *n* (%)	137 (35.0)	45 (37.2)	92 (34.1)	0.314
Diabetes, *n* (%)	56 (14.3)	14 (11.6)	42 (15.6)	0.189
Coronary artery disease, *n* (%)	29 (7.4)	11 (9.1)	18 (6.7)	0.258
Prolonged bed rest^#^, *n* (%)	24 (6.1)	4 (1.5)	20 (16.5)	<0.001
Tumor stage				
Stage I, *n* (%)	25 (6.4)	4 (3.3)	21 (7.8)	0.002
Stage II, *n* (%)	69 (17.6)	13 (10.7)	56 (20.7)	
Stage III, *n* (%)	73 (18.7)	18 (14.9)	55 (20.4)	
Stage IV, *n* (%)	224 (57.3)	86 (71.1)	138 (51.1)	
Urban households, *n* (%)	245 (62.7)	80 (66.1)	165 (61.1)	0.203
KPS scores ≥90, *n* (%)	212 (54.2)	50 (41.3)	162 (60.0)	<0.001
Surgical orifice fistulae, *n* (%)	48 (12.3)	17 (14.0)	31 (11.5)	0.288
History of surgery within 12 months, *n* (%)	224 (57.3)	71 (58.7)	153 (56.7)	0.398
Pain medication use, *n* (%)	103 (26.3)	44 (36.4)	59 (21.9)	0.002
NRS-2002 scores ≥ 3	130 (33.2)	90 (74.4)	40 (14.8)	<0.001
BMI (kg/m^2^)	21.54 ± 3.43	17.98 ± 2.26	22.84 ± 2.80	<0.001
Laboratory indicators				
RBC (×10^12^/L)	4.17 (3.54, 4.65)	3.67 (3.03, 4.18)	4.34 (3.79, 4.78)	<0.001
WBC (×10^9^/L)	5.69 (4.17, 8.03)	6.86 (4.51, 9.81)	5.40 (4.01, 7.37)	0.001
PLT (×10^9^/L)	193.00 (142.00, 267.00)	196.00 (142.25, 252.75)	189.00 (142.25, 252.75)	0.157
HGB (g/L)	128.00 (108.00, 146.00)	112.00 (93.00, 130.00)	134.00 (119.00, 149.25)	<0.001
ALB (g/L)	36.63 ± 6.01	32.56 ± 6.02	38.46 ± 5.03	<0.001
PAB (g/L)	161.59 ± 59.25	127.00 ± 52.97	174.08 ± 56.60	<0.001
ALT (u/L)	21 (14.00, 34.00)	19.00 (12.00, 35.00)	22.00 (15.00, 34.00)	0.041
AST (u/L)	26.00 (19.00, 40.00)	25.00 (17.00, 49.00)	26.00 (20.00, 38.75)	0.584
Urea (mmol/L)	5.22 (4.20, 6.99)	5.70 (4.37, 9.04)	5.17 (4.07, 6.57)	0.002
Cr (mmol/L)	68.15 (58.38, 80.50)	69.00 (55.35, 83.55)	68.00 (60.00, 80.00)	0.612
NLR	3.37 (1.96, 6.442)	5.02 (3.06, 9.10)	2.69 (1.78, 4.35)	<0.001
SII index	600.00 (315.40, 1,245.84)	936.34 (493.96, 2,270.34)	499.46 (283.71, 1,016.22)	<0.001
PLR	161.61 (109.24, 242.96)	198.52 (124.60, 309.70)	147.54 (105.73, 213.90)	<0.001
PNI	42.83 ± 7.83	37.84 ± 7.31	45.07 ± 6.24	<0.001
NRS-2002 score, *n* (%)				
0–2	261 (66.8)	31 (25.6)	230 (85.2)	<0.001
≥3	130 (33.2)	90 (74.4)	40 (14.8)	

^#Whether chronically bedridden was defined as 72 h of continuous quiet bed rest or at least seven consecutive days of >4 h per day in addition to sleep time.^

RBC, red blood cell; WBC, white blood cell; PLT, platelet; HGB, hemoglobin; ALB, albumin; PAB, prealbumin; ALT, alanine aminotransferase; AST, aspartate aminotransferase; Urea, urea; Cr, creatinine; NLR, neutrophil-to-lymphocyte ratio; SII, systemic immune-inflammation index; PLR, platelet-to-lymphocyte ratio; PNI, prognostic nutritional index.

**Figure 2:**
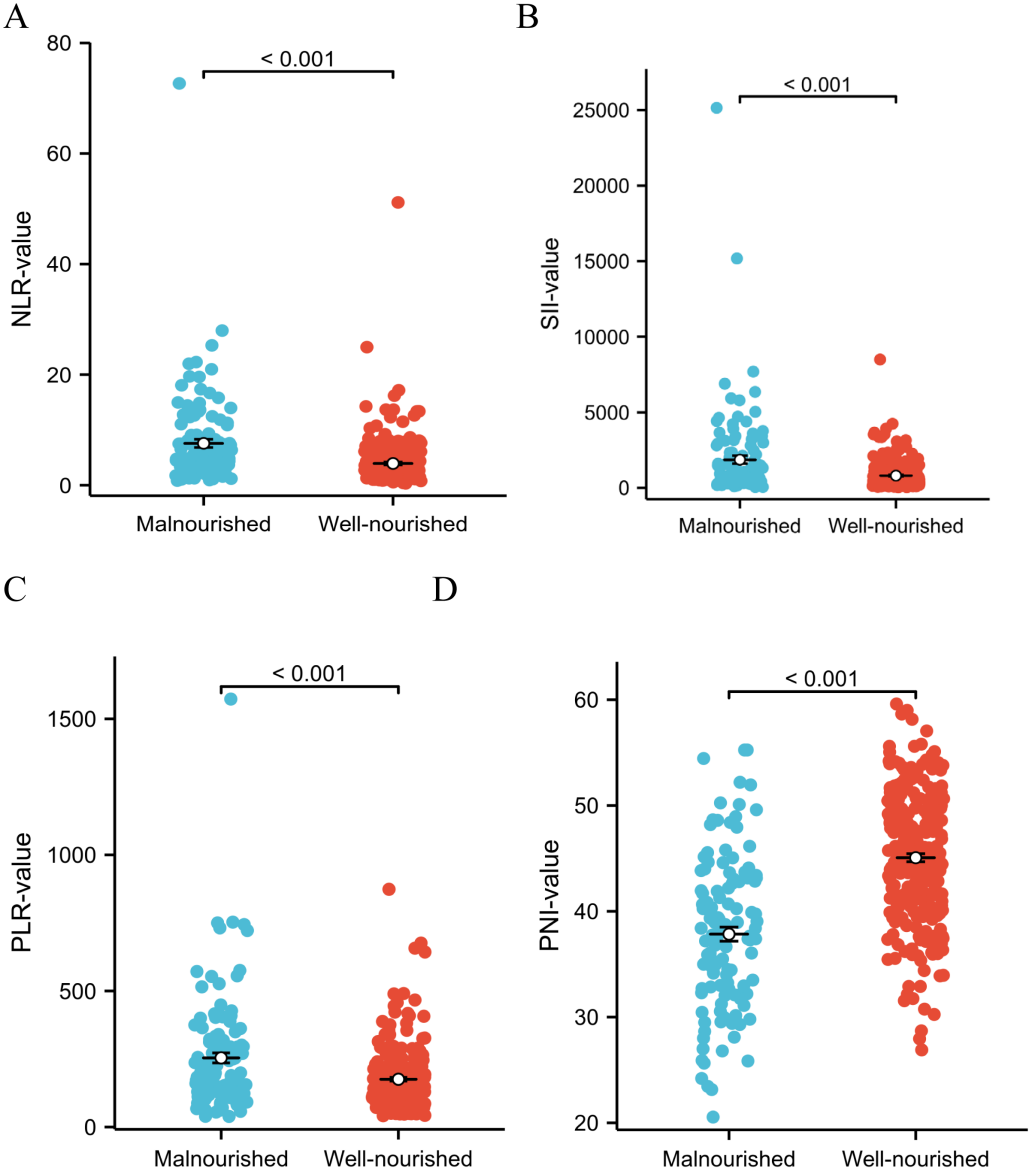
(A–D) Analyzing the different indicators as continuous variables revealed significant differences in NLR index, SII, PLR, and PNI between the malnutrition group and the well-nourished group (All *p* < 0.001). Statistical analysis was performed using the Student’s t-test.

**Figure 3:**
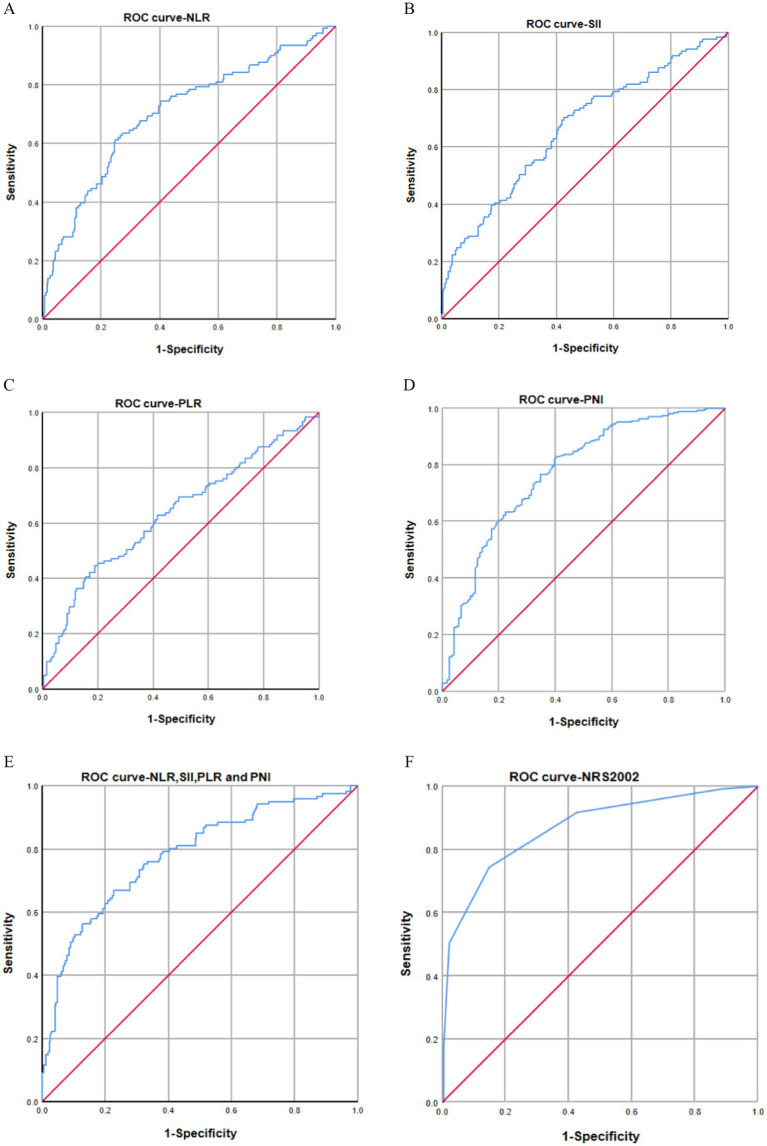
(A–F) AUC curves for predicting malnutrition using NLR (A), SII (B), PLR (C), PNI (D), combined diagnostics (NLR, SII, PLR, and PNI) (E), and NRS-2002 (F). The AUC values indicated that NLR index (AUC: 0.705, 95% CI: 0.647–0.762), SII (AUC: 0.665, 95% CI: 0.606–0.725), PLR (AUC: 0.636, 95% CI: 0.647–0.762), PNI (AUC: 0.773, 95% CI: 0.722–0.825), and combined diagnosis (AUC: 0.784, 95% CI: 0.735–0.833) had AUC values below that of NRS-2002 (AUC: 0.868, 95% CI: 0.828–0.909).

**Figure 4:**
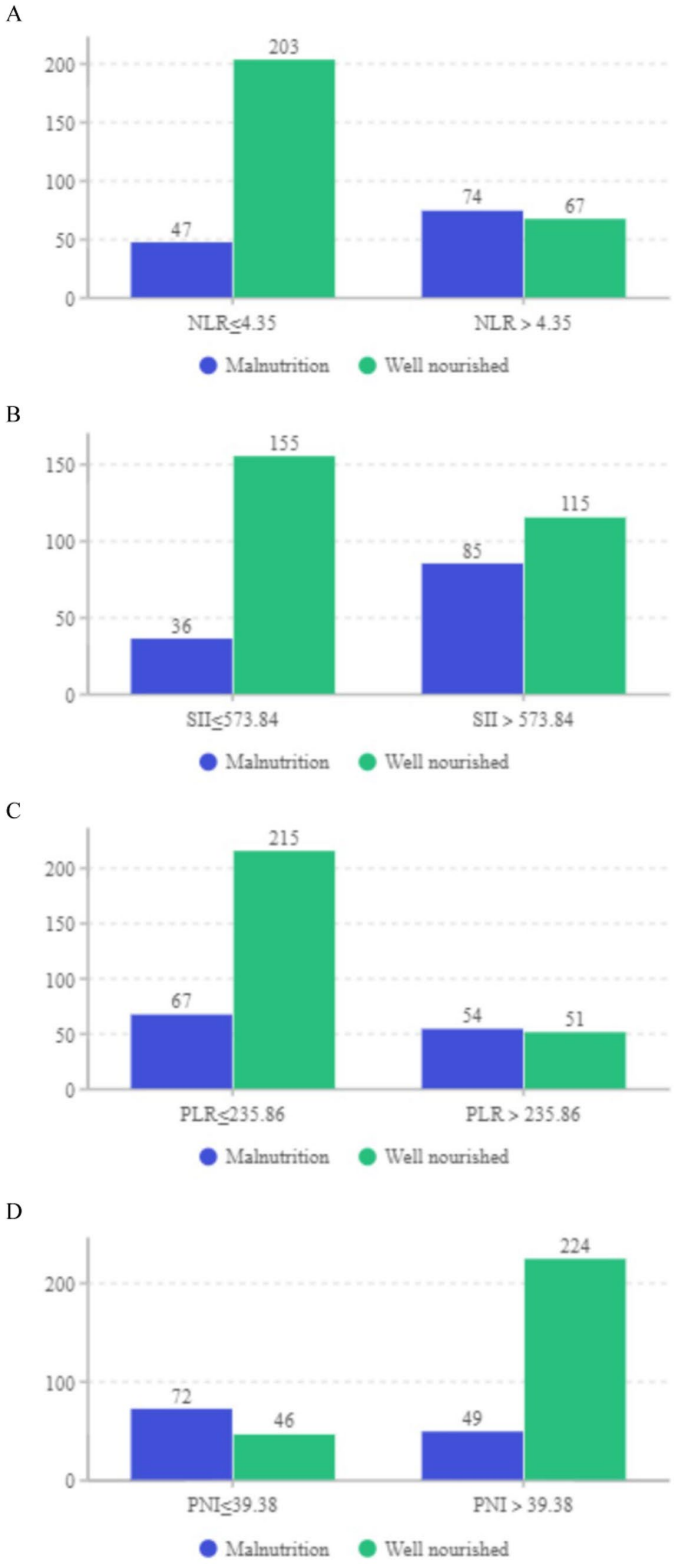
(A–D) Distribution of well-nourished and malnourished patients based on cut-off values for NLR, SII, PLR, and PNI (All *p* < 0.05).

Our study focused on analyzing the NLR, SII index, PLR, and PNI parameters as continuous variables, revealing statistically significant differences between malnourished and well-nourished groups (Figures 2A–D, *p* < 0.001). Additionally, we conducted ROC curve analysis to assess the predictive ability of NLR, SII index, PLR, PNI, and the combined metrics for malnutrition, as well as evaluating the predictive capacity of NRS-2002 (Figures 3A–E). Analysis of the AUC values indicated that NLR, PNI, and the combined diagnostics (NLR, SII, PLR, and PNI) exhibited superior predictive capabilities for malnutrition. Further details regarding AUC values, cut-off points, sensitivity, and specificity for predicting malnutrition occurrence can be found in Table 2. Furthermore, following the calculation of cut-off values for the pertinent indicators, bar graphs were utilized to evaluate the discriminatory capacity of said indicators. Within the scope of the research, all participants were categorized into two groups based on these cut-off values, with further division into malnourished and well-nourished subgroups. A notable prevalence of malnourished colorectal cancer patients was observed in the categories of elevated NLR, SII index, and PLR levels, as well as decreased PNI levels (Figures 4A–D). The chi-square *p*-value for the comparison between the malnourished group and well-nourished group was <0.05.

**Table 2:** AUC, cut-off value, sensitivity and specificity for predicting the occurrence of malnutrition in CRC patients.

Variables	Cut-off value	AUC (95% CI)	Sensitivity	Specificity	PPV	NPV
NLR	4.350	0.705	0.610	0.750	0.740	0.570
SII	573.84	0.665	0.700	0.570	0.680	0.590
PLR	235.86	0.636	0.450	0.810	0.750	0.500
PNI	39.380	0.773	0.590	0.830	0.800	0.620

AUC, area under receiver operating characteristic; NLR, neutrophil-to-lymphocyte ratio; SII, systemic immune-inflammation index; PLR, platelet-to-lymphocyte ratio; PNI, prognostic nutritional index; CRC, colorectal cancer; PPV, Positive Predictive Value; NPV, Negative Predictive Value.

**Figure 5:**
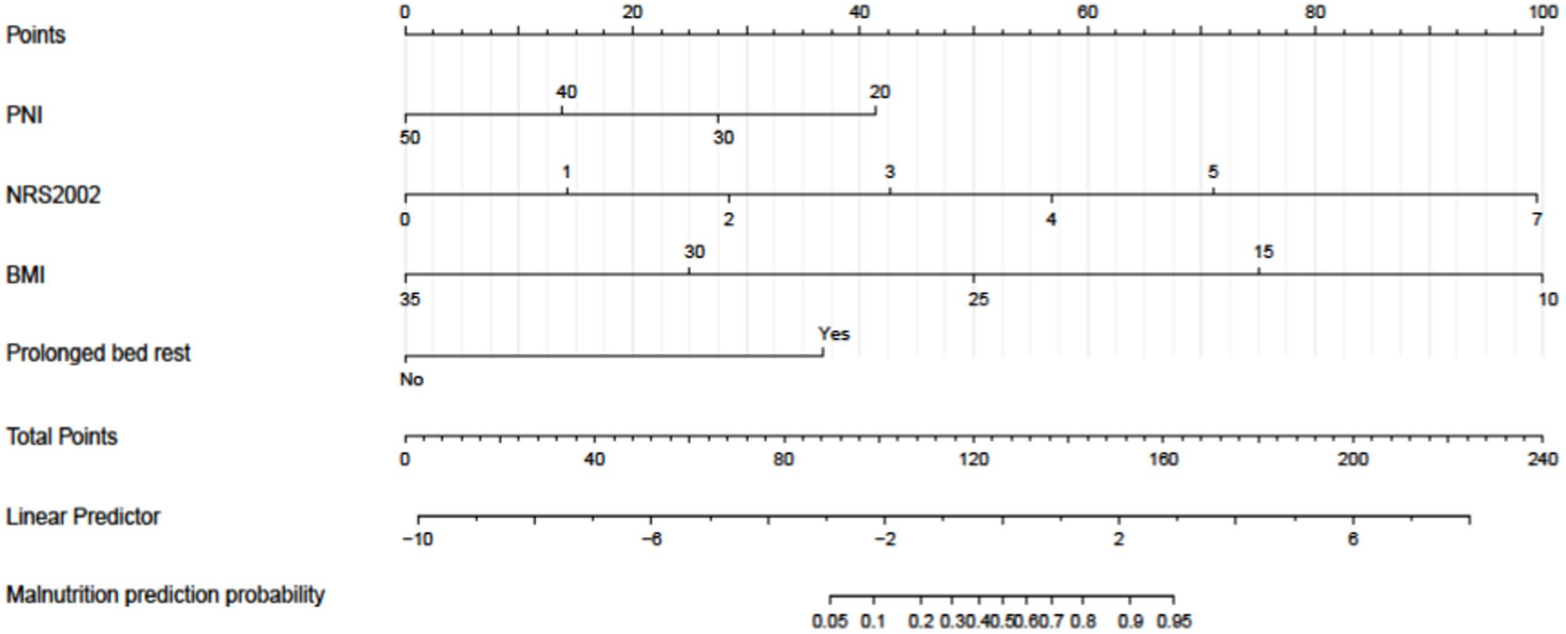
A nomogram model was developed to predict the risk of malnutrition in patients diagnosed with colorectal cancer (CRC). The nomogram utilizes prediction variables to assign points, which are then totaled to calculate a cumulative score. This score corresponds to the predicted risk of malnutrition in the patient.

The study was further enhanced by incorporating all relevant variables for multivariate analysis, which consistently demonstrated that NRS-2002, prolonged bed rest, BMI, and PNI were significant independent risk factors for malnutrition development in CRC patients (Table 3). A risk prediction model was subsequently constructed using binary logistic analysis, resulting in a nomogram that included the aforementioned four significant factors (Figure 5). The logistic model confirmed that all variables were significant predictors of malnutrition onset. The nomogram analysis revealed that BMI and NRS-2002 were the most significant predictors of malnutrition, with PNI and Prolonged bed rest also playing a role. Detailed annotations are provided to explain the utilization of the nomogram. The model’s Hosmer-Lemeshow test yielded a value of 13.633 with a corresponding *p* value of 0.34.

**Table 3:** Multiple logistic regression analysis of CRC patients with malnourished.

Variables	OR	95% CI	*p* value
NRS-2002			<0.001
0–2	Reference		
≥3	7.994	3.436–18.599	
Prolonged bed rest			<0.001
No	Reference		
Yes	14.285	3.312–61.617	
BMI (Chinese Standard, kg/m^2^)			<0.001
<18.5	Reference		
≥18.5, <24	0.009	0.002–0.034	
≥24	0.001	0.000–0.013	
PNI			<0.001
≤39.38	Reference		
>39.38	0.144	0.061–0.339	

NRS-2002, Nutritional Risk Screening 2002; BMI, Body Mass Index; Prolonged bed rest, 72 consecutive hours of quiet bed rest or at least seven consecutive days of sleep greater than 4 h per day; PNI, prognostic nutritional index; CRC, colorectal cancer. NRS-2002: The NRS scores of 1–2 indicate a low nutritional risk group, while scores of ≥3 indicate a high nutritional risk group. BMI: Calculated according to the Asian classification standards. Prolonged bed rest: Defined as being chronically bedridden for 72 h of continuous quiet bed rest or at least seven consecutive days of more than 4 h per day in addition to sleep time. PNI: Evaluated using the optimal cut-off value derived from the analysis.

**Figure 6:**
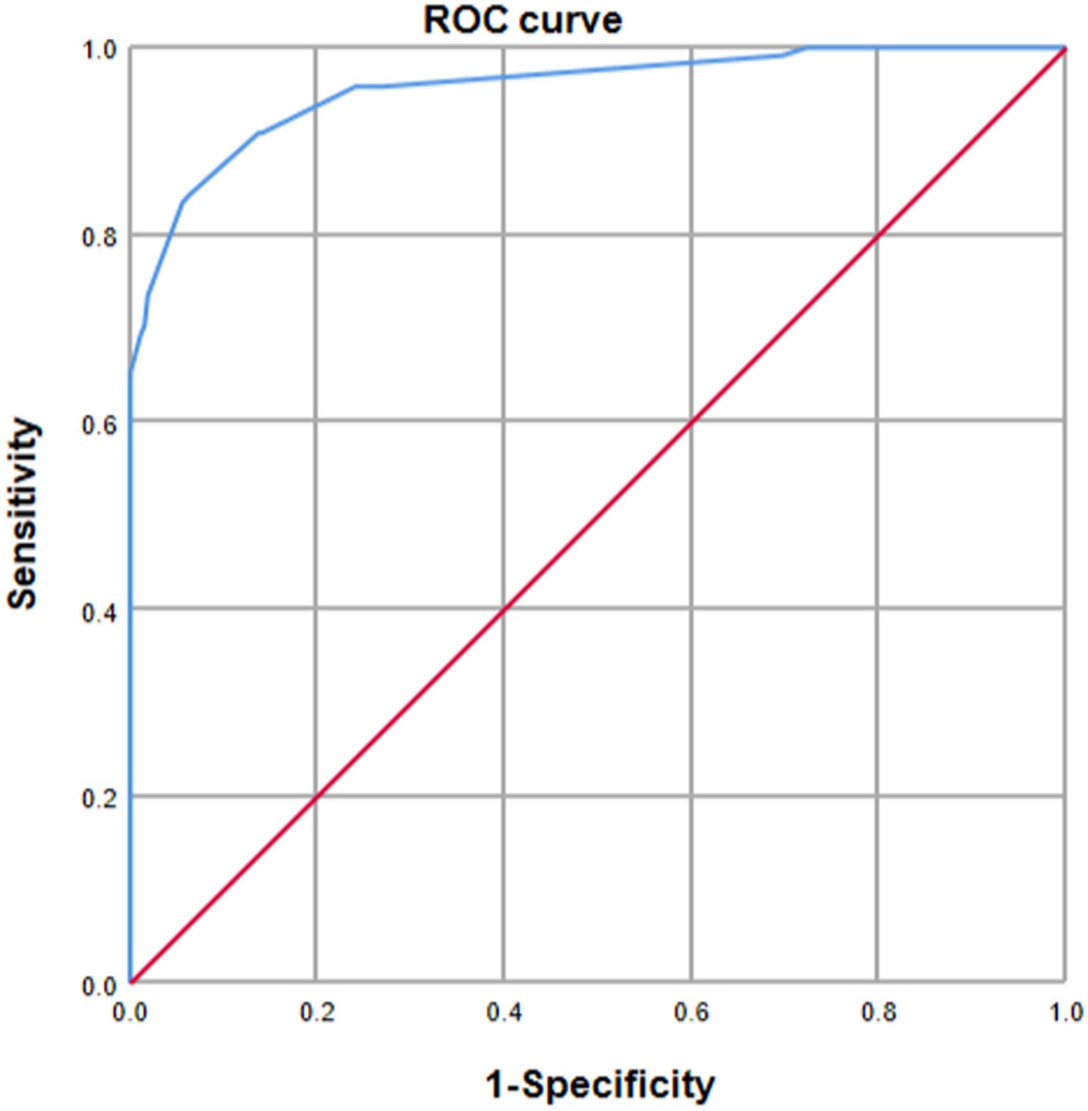
Receiver operating characteristic (ROC) curve analysis was utilized to evaluate the predictive performance of the nomogram for malnutrition risk in patients diagnosed with colorectal cancer (CRC). The AUC was calculated through bootstrapping, with the 95% confidence interval (CI) estimated. The two-sided *p*-value was determined. The AUC was found to be 0.958 (95% CI, 0.937–0.979).

**Figure 7:**
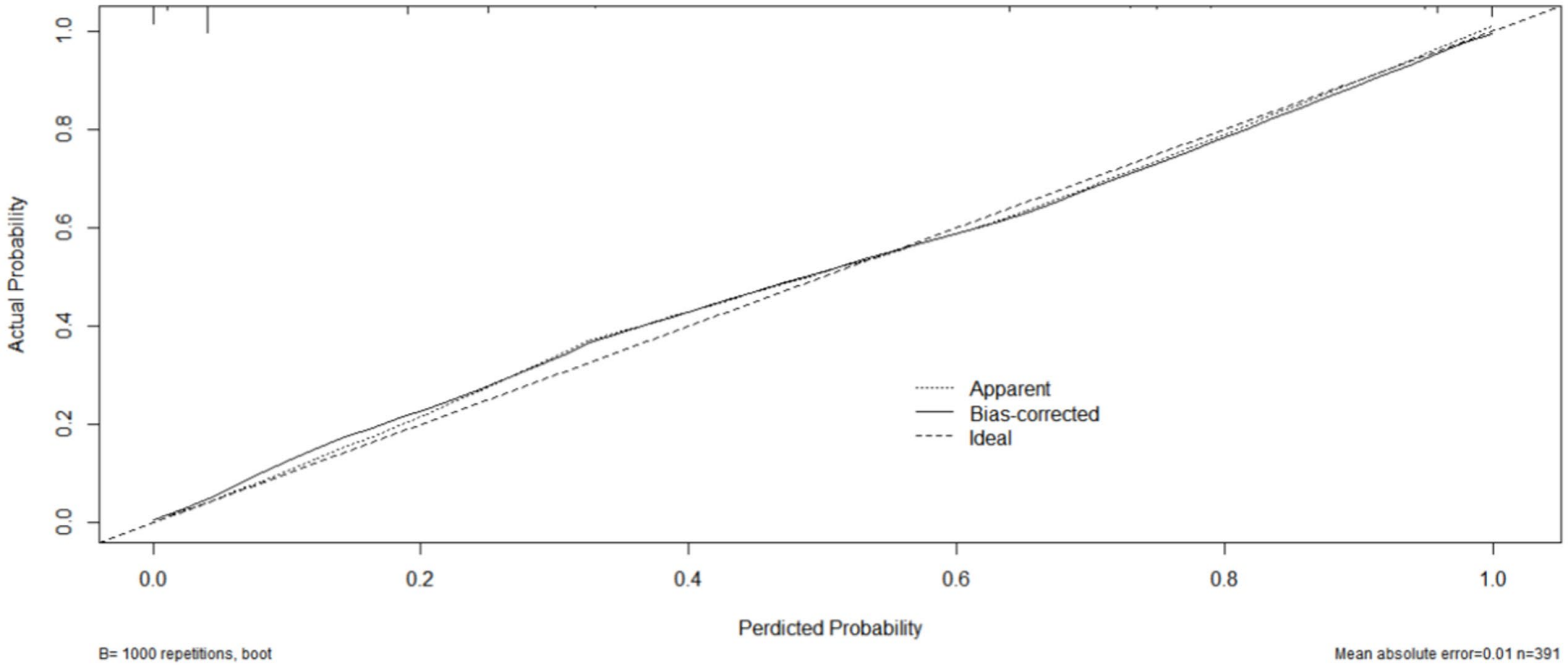
The calibration curve of the nomogram model for predicting malnutrition occurrence in colorectal cancer (CRC) patients. The gray thick line represents the ideal model’s perfect prediction, the black dashed line signifies the target parameter, and the solid black line illustrates the model’s performance. Bootstrap resampling was conducted 1,000 times.

**Figure 8:**
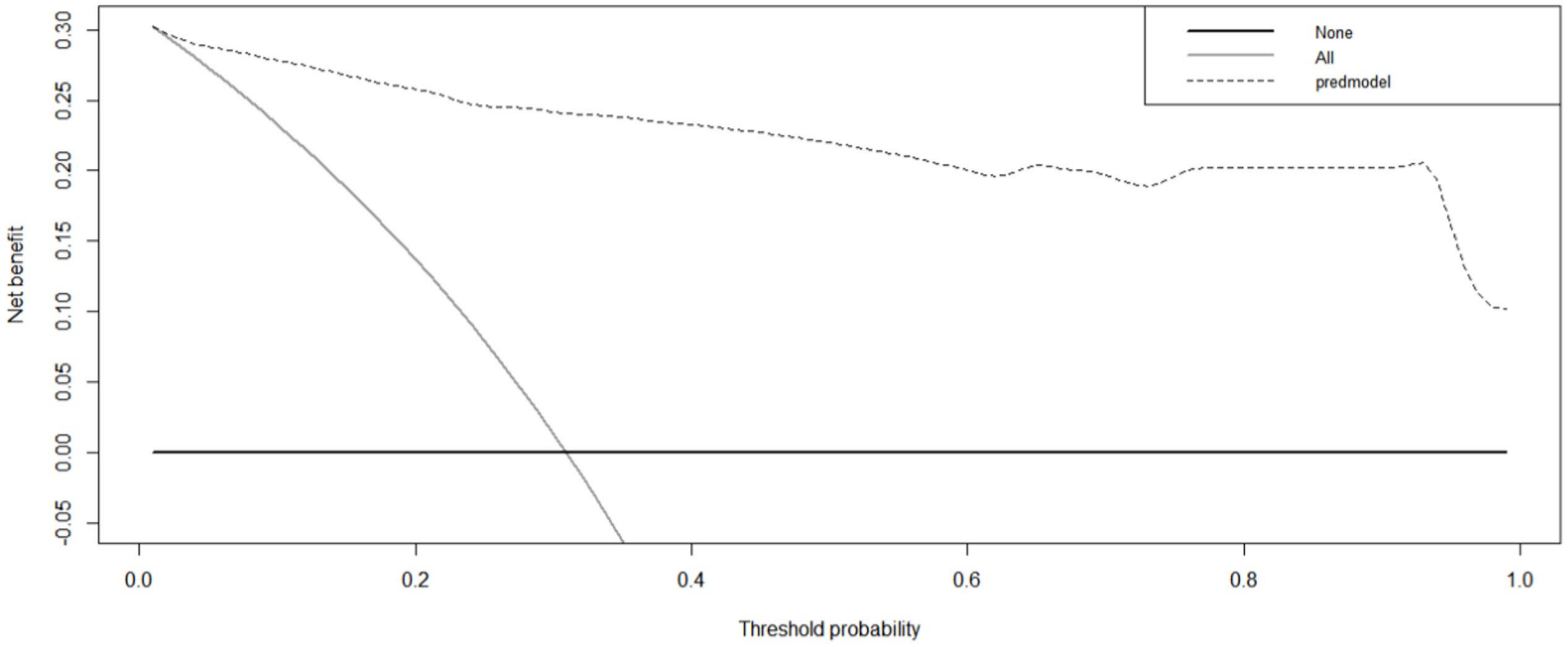
The clinical decision curve of the nomogram for predicting malnutrition in colorectal cancer (CRC) patients. The prediction model is denoted by a black dashed line, with the gray solid line representing samples that received intervention and the black solid horizontal line representing samples that did not receive intervention. The graph visually represents the expected net benefit for each patient based on the nomogram’s predictive accuracy for malnutrition. As the model curve extends, the net benefit increases, indicating improved predictive ability.

Internal validation using the bootstrap validation method demonstrated a C-index of 0.958, suggesting that the developed prediction model exhibits strong predictive performance. Subsequent ROC analysis was conducted to assess the predictive accuracy of the nomogram model for malnutrition (Figure 6). The AUC value for the nomogram was determined to be 0.958 (95% CI, 0.937–0.979), indicating superior discriminatory capability compared to the results presented in Figure 3F. The ROC analysis revealed a sensitivity of 0.843 and specificity of 0.937, resulting in a cut-off value of 0.78, further emphasizing the model’s robustness in identifying at-risk patients. Additionally, our study demonstrated a strong correlation between predicted and observed probabilities through calibration curves (Figure 7), and further assessed the clinical utility of the malnutrition nomogram by conducting a clinical DCA (Figure 8). Utilizing our predictive nomogram for visual prediction of the dependent variable formation may offer greater benefits compared to categorizing all patients as either low or high risk. The predictive model achieved a PPV of 85% and a NPV of 90%, indicating its strong accuracy in diagnosing malnutrition among CRC patients. While utilizing our predictive nomogram for visual prediction of the dependent variable may provide a more nuanced assessment of risk compared to categorizing all patients as either low or high risk, further research is needed to fully validate the benefits of this approach.

## 4 Discussion

Malnutrition poses a prevalent and significant risk in hospitalized patients with malignant tumors, particularly in those with CRC as a result of inherent physiological processes, unique anatomical considerations, and additional contributing factors that collectively elevate the likelihood of malnutrition compared to patients with non-gastrointestinal malignancies (20). In our investigation, a total of 121 (30.95%) inpatients diagnosed with CRC were found to be malnourished, a prevalence consistent with the range of 20–37% reported in existing literature (2). However, our findings diverge significantly from the 87% malnutrition rate cited in certain contemporary sources. Discrepancies in prevalence rates may be attributed to variations in the screening instruments utilized across studies, as well as the specific patient population under examination. Notably, our study exclusively focused on a subgroup of CRC patients characterized by favorable overall health and extended life expectancy, while excluding individuals with a poorer prognosis.

The findings of the risk factor analysis conducted in our study indicate that malnourished colorectal cancer patients exhibit advanced age, primarily attributable to diminished daily functional capacity, heightened complications, and impaired nutrient absorption in older individuals. Our results align with existing literature demonstrating a positive association between age and the prevalence of malnutrition (21, 22). Furthermore, upon further analysis, it was determined that Karnofsky Performance Status (KPS) score, pain medication use, RBC, WBC, HGB, ALB, PAB, ALT and Urea levels were identified as the primary risk factors for malnutrition in patients diagnosed with CRC. KPS score is commonly utilized to assess the functional capacity of cancer patients, with lower scores indicating greater impairment (23). Consequently, individuals with lower KPS scores are more likely to experience complications, reduced quality of life, prolonged periods of bed rest, and ultimately, poorer nutritional status (24). In our investigation, we observed a noteworthy phenomenon wherein oral pain medications contribute to the occurrence of malnutrition. This phenomenon could be attributed to the impact of oral pain medications commonly prescribed for cancer pain management on patients’ appetite, potentially leading to reduced food intake. Alternatively, it could be linked to the adverse effects of painkillers, such as gastrointestinal ulcers or bleeding, which may impair digestive and absorptive functions, consequently affecting nutrient absorption. Furthermore, inadequate pain management in certain individuals receiving oral pain medication may impede their oral food intake, particularly among oncology patients, thereby influencing the onset of malnutrition (25). Numerous studies (26–29) have identified RBC, HGB, ALB, and PAB as significant indicators of malnutrition. Our findings further support the notion that decreased levels of RBC, HGB, ALB, and PAB are associated with an increased risk of malnutrition. This relationship has been widely acknowledged by medical researchers, both theoretically and empirically.

In recent years, a significant number of researchers have examined inflammatory biomarkers, primarily in the context of oncological prognosis (13). Some scholars have also explored their relationship with malnutrition, particularly in non-oncological patients (5, 8, 16). To the best of our knowledge, this study represents the first exploration of the predictive value of inflammatory markers for malnutrition in hospitalized patients with colorectal cancer. In our analysis of the four common inflammatory indices, we observed that NLR, SII index, and PLR levels were significantly lower in well-nourished individuals compared to malnourished patients, whereas the PNI displayed an opposite trend. Utilizing specific cut-off values as categorical variables, we determined that the likelihood of malnutrition was notably reduced when NLR levels were below 4.35, SII index levels were below 573.84, PLR levels were below 235.86, and PNI levels were above 39.38. Subsequent examination revealed that the NRS-2002 screening tool alone was equally effective in predicting malnutrition in CRC patients when compared to the combined diagnosis (NLR, SII, PLR and PNI) based on the inflammatory indices. Numerous studies have utilized the SII index as a primary indicator of malnutrition (8, 30). However, our study conducted a comparative analysis of NLR, SII index, PLR, and PNI revealing that NLR and PNI were more effective predictors of malnutrition than SII index and PLR. These findings align with the conclusions drawn by Tezcan Kaya and Rui-Hong Wang’s research teams (6, 16). The PNI utilizes serum albumin concentration and lymphocyte count (31), established prognostic markers of immune function, either independently or in conjunction with other inflammatory markers, to offer a reliable and objective evaluation of nutritional status and mitigate negative health outcomes (32). This index has been extensively embraced as a valuable predictive tool for identifying malnutrition across various types of cancer (33, 34), and our study further validates the predictive utility of the PNI in this context, which may allow a wide range of researchers to take full advantage of this useful biomarker.

Although the conclusions of our experiments are very sensational, the etiology of malnutrition in oncology patients remains unclear. Numerous studies indicate that tumors are often associated with a persistent inflammatory response (35), which may contribute to insufficient nutritional intake by affecting nociceptors and the endocrine system (36). Prolonged immune activation may lead to heightened nutritional requirements, while chronic inadequate intake can impair both innate and adaptive immune responses, thereby increasing vulnerability to illness and worsening malnutrition (8, 36, 37). Therefore, the interconnectedness of immune function, inflammation, and malnutrition forms a cyclical relationship. Our study supports the utilization of systemic inflammatory biomarkers for evaluating malnutrition.

Presently, prevalent clinical malnutrition risk screening tools such as MNA (Mini Nutritional Assessment) and Nutritional Risk Screening 2002 (NRS-2002), while widely utilized by healthcare professionals, are constrained in their precision and dependability due to the insufficient level of evidence derived from expert consensus, subjective elements, and uncertainty (38, 39). Although, our present investigation focused on exploring the correlation between the four indices and malnutrition in patients with CRC, and the results were satisfactory, however, we, in order to further enrich our experimental study, we, in order to further optimize the current malnutrition prediction tool, we analyzed it by multivariate logistic regression analysis, which resulted in the discovery of the important factors for the prediction of malnutrition: prolonged bed-riddenness, BMI, NRS-2002 and PNI, and recombined these four main factors, which are clinically accessible and do not add additional healthcare costs to patients for malnutrition prediction to form a new predictive nomo model, which showed surprising and satisfactory clinical performance in both calibration curves and DCA analyses, which is a bold attempt. To the best of our knowledge, this is the first initial validated instrument that merges a risk assessment tool with an inflammation index for optimal amalgamation in forecasting the onset of malnutrition in hospitalized patients with colorectal cancer.

Our research indicates that it is advisable to assess patients with colorectal cancer for biomarkers such as NLR, SII index, PLR, and PNI promptly upon admission. It is evident that timely intervention is necessary for CRC patients who are chronically bedridden, at high risk of malnutrition as indicated by the NRS-2002 score at high risk, have a low BMI, and exhibit a poor PNI during inpatient hospitalization. This warrants heightened awareness and vigilance among healthcare providers and caregivers. Nevertheless, it is important to acknowledge the limitations of our study. Specifically, our study offered a thorough evaluation of the nutritional status of the participants, key nutritional indicators such as unintentional weight loss, triceps skinfold thickness, and waist circumference were not fully incorporated into the analysis. The potential presence of bias in the criteria used to assess malnutrition, including subjective factors like reduced food intake or absorption, may have influenced the outcomes of our study. It is imperative to note that the constraints of our retrospective study conducted in a single center necessitate the validation of our findings through data from multicenter, prospective studies.

## 5 Conclusion

Our study findings indicate that NLR, SII index, PLR, and PNI serve as predictive indicators for malnutrition in hospitalized patients with CRC, thus enhancing the effectiveness of malnutrition screening and potentially informing targeted intervention strategies. Additionally, BMI, extended periods of bed rest, elevated NRS scores, and PNI parameters were found to be correlated with malnutrition. Through the application of multiple regression analysis, risk factors for malnutrition in elderly patients with malignant CRC were identified. A clinical prediction model was developed, calibrated, and validated, exhibiting robust accuracy and clinical relevance for prognostication, thereby facilitating the implementation of efficacious early intervention measures to mitigate the risk of malnutrition.

## Data Availability

The raw data supporting the conclusions of this article will be made available by the authors without undue reservation.
